# Comparison of ANN and XGBoost surrogate models trained on small numbers of building energy simulations

**DOI:** 10.1371/journal.pone.0312573

**Published:** 2024-10-25

**Authors:** Sanja Stevanović, Husain Dashti, Marko Milošević, Salem Al-Yakoob, Dragan Stevanović

**Affiliations:** 1 Mathematical Institute of the Serbian Academy of Sciences and Arts, Belgrade, Serbia; 2 FAMNIT, University of Primorska, Koper, Slovenia; 3 College of Architecture, Kuwait University, Safat, Kuwait; 4 Faculty of Sciences and Mathematics, University of Niš, Niš, Serbia; 5 Faculty of Science, Kuwait University, Safat, Kuwait; 6 Abdullah Al Salem University, Khaldiya, Kuwait; Newcastle University, UNITED KINGDOM OF GREAT BRITAIN AND NORTHERN IRELAND

## Abstract

Surrogate optimisation holds a big promise for building energy optimisation studies due to its goal to replace the use of lengthy building energy simulations within an optimisation step with expendable local surrogate models that can quickly predict simulation results. To be useful for such purpose, it should be possible to quickly train precise surrogate models from a small number of simulation results (10–100) obtained from appropriately sampled points in the desired part of the design space. Two sampling methods and two machine learning models are compared here. Latin hypercube sampling (LHS), widely accepted in building energy community, is compared to an exploratory Monte Carlo-based sequential design method mc-intersite-proj-th (MIPT). Artificial neural networks (ANN), also widely accepted in building energy community, are compared to gradient-boosted tree ensembles (XGBoost), model of choice in many machine learning competitions. In order to get a better understanding of the behaviour of these two sampling methods and two machine learning models, we compare their predictions against a large set of generated synthetic data. For this purpose, a simple case study of an office cell model with a single window and a fixed overhang, whose main input parameters are overhang depth and height, while climate type, presence of obstacles, orientation and heating and cooling set points are additional input parameters, was extensively simulated with EnergyPlus, to form a large underlying dataset of 729,000 simulation results. Expendable local surrogate models for predicting simulated heating, cooling and lighting loads and equivalent primary energy needs of the office cell were trained using both LHS and MIPT and both ANN and XGBoost for several main hyperparameter choices. Results show that XGBoost models are more precise than ANN models, and that for both machine learning models, the use of MIPT sampling leads to more precise surrogates than LHS.

## 1 Introduction

Design of contemporary energy efficient buildings relies in large part on simulations of their energy behaviour. To optimise various building properties it is often necessary to employ general optimisation methods, such as genetic algorithms, which may need to perform simulations of hundreds or thousands of building variants to identify optimal combinations of their parameters. Depending on the complexity of building properties in question, such simulations can last from a few seconds to several hours, especially when it comes to demanding daylighting or computational fluid dynamics simulations. Even in cases when a single simulation lasts only a few seconds, it may happen that there are tens of parameters with several different feasible values each, so that tens of thousands of simulations might be necessary to arrive at optimal parameter combinations. In such situations surrogate optimisation becomes a worthwhile alternative that can often reach close to optimal solutions in a fraction of the time.

Surrogate optimisation, also known as black-box optimisation, uses machine learning (ML) methods to create a surrogate model that will predict results of future simulations from the results of existing simulations. This surrogate model, which may predict thousands of simulation results in the time needed to perform a single new actual simulation, is iteratively optimised and improved by balancing two different perspectives: exploitation, which focuses on identifying optimal solutions of the current surrogate model, and exploration, which focuses on sampling points from the unexplored areas of the design space, understanding that the surrogate model predictions will be most precise in the vicinity of already simulated points. This exploitation-exploration conundrum selects one or several new parameter combinations for full simulation, whose results are then used to retrain the surrogate model and repeat the above procedure.

While the choices of the optimisation method and the way of balancing exploration and exploitation are often the main focus of surrogate optimisation [1], the ML method used to train the surrogate model and the sampling method used to select the initial building variants for simulation represent its essential parts and it is important that they are well chosen in the first place, as the surrogate models should be trained quickly and with high prediction accuracy. These two latter steps are often used independently in building energy literature to create global surrogate models, which also enable one to perform sensitivity and uncertainty analyses, to either identify the most important input parameters of the building model or estimate how the uncertainty in input parameters may propagate onto the simulation (surrogate) outcomes.

Westermann and Evins [2] give a comprehensive review of building design research studies that use surrogate modelling for predicting aggregated design metrics (such as annual energy use) in either conceptual design, sensitivity analysis, uncertainty analysis or building design optimisation. They indicate that Latin hypercube sampling (LHS) is the most applied sampling scheme, while multiple linear regression (LR) is still the most popular surrogate model type in building related literature. However, they also point out the disadvantage of multiple LR which has to assume that the functional relationship between the inputs and the outputs is known a priori which is not necessary for modern ML methods such as artificial neural networks (ANN), Gaussian process models (GP), support vector regression (SVR), multivariate adaptive regression splines (MARS), radial basis function (RBF) networks, random forests (RF), or model ensembles, since these create surrogate models with higher prediction accuracies a posteriori by fitting the observed input-output value pairs into their much more general frameworks.

Roman et al. [3] review the use of surrogate modelling in building energy literature, observing that researchers mostly use ANN, GP, polynomial regression, SVM, LR and MARS. Their review points out that, over the last decade, ANN has overtaken GP and SVM as favourite building performance simulation (BPS) surrogate modelling method, partly due to its favourable trade-off between accuracy and computational cost. Vast majority of ANNs used in reviewed BPS studies are feed-forward NNs with one hidden layer, with only a small percent of researchers opting to use feed-forward NNs with 2–4 or more hidden layers or to use other types of NNs. In their review, Roman et al. [3] also confirm that LHS is the most used sampling method in building energy community.

Observing that ANNs have become the most favoured surrogate modelling method among BPS researchers in recent years, Lu et al. [4] give an extensive review of the applications of ANNs in more than 300 BPS studies published since 2016. They provide detailed guidance on 12 different types of ANNs used in BPS literature, which belong to the general categories of feed-forward, recurrent and convolutional NNs. It is observed that feed-forward NNs make up the majority of papers and usually model dependencies between energy-related building features and energy data, while recurrent NNs and convolutional NNs, which are better suited for predicting time series building energy data, started to gain traction in more recent studies.

While widely available graphical processing units enabled training of highly complex ANN models during the last decade, whose capabilities raised public interest and, consequently, led to the proliferation of ANNs in all fields of science and technology, it was independently observed that, in the case of regression with smaller number of available input-output data pairs, ensemble models more easily reach higher accuracy and usually generalise better than single models, including ANNs [5]. Ensemble models iteratively train a set of simple base models, known as weak learners, and combine them together to create a more predictive model. Among them, gradient-boosted regression trees [6] (GBRT) quickly gained popularity in ML community. Papadopoulos et al. [7] provide one of the earliest evaluations of GBRT performance in predicting heating and cooling loads for 12 buildings from the data set of Tsanas and Xifara [8], which have the same volume, but varying relative compactness. Their study showed that GBRT significantly outperforms GP, SVR, RF and a SVR/ANN ensemble for both heating and cooling loads. More recent notable example of the applicability of GBRT models in BPS studies can be found in the recent ASHRAE Great Energy Predictor III competition [9], which provided over 20 million of metered hourly energy data points from university, municipal and healthcare buildings around the world as training data and asked for the most accurate surrogate model to predict over 41 million of metered test data points. As it turned out, the first five best solutions all used ensembles that predominantly relied on GBRT methods, which were in some cases enlarged with NNs.

XGBoost [10] is a well-known recent GBRT variant that uses Newton-Raphson boosting to iteratively build an ensemble of regression trees. It already became a surrogate model of choice for structured data in many ML competitions [11], and it was used in several other BPS studies as well. Fan et al. [12] compare several surrogate modelling methods in predicting 24h ahead building cooling load profiles for an educational building in Hong Kong. Allowing ANNs to have between one and ten hidden layers and either rectified linear unit (ReLU), hyperbolic tangent or sigmoid as an activation function, they observe that for their amount of available data optimal choice of ANN has two hidden layers and ReLU as the activation function. Nevertheless, XGBoost turns out to be the superior method over ANN and SVR in their study, both in terms of the prediction quality and the computational cost. Touzani et al. [13] train surrogate models on high frequency metered electricity consumption data of commercial buildings, and show that XGBoost achieves higher accuracy than RF and piecewise LR in predicting electricity consumption time series. Wang et al. [14] further conclude that XGBoost exhibits optimal efficiency, when compared to ANNs, SVR and RF, in predicting municipal hourly heating energy consumption of a residential quarter in Tianjin, China. Song et al. [15] develop surrogate models to predict global solar radition from meteorological measurements. For model training they use solar radiation and meteorological data measured since 1993 at 130 locations in China, and then apply developed models to predict solar radiation and assess photovoltaic power potential for further 2,474 locations across China, where only meteorological data are measured. They show that XGBoost with default values of its hyperparameters already outperforms LightGBM, ANN, SVR with radial basis function kernel and *k*-nearest neighbor algorithm, but also that proper optimisation of XGBoost hyperparameters may further improve its prediction quality. Robinson et al. [16] train surrogate models for predicting annual commercial building energy consumption on the available sets of measured energy consumption data in USA. Their study shows that, over all classes of commercial buildings, XGBoost predictions outperform predictions of other surrogate models, which include ANNs with one hidden layer, SVR, RF and LR.

The above studies clearly suggest that ANNs have become most popular surrogate models in building energy community, while at the same time XGBoost, recommended by the ML community, offers better performance, both in prediction quality and computational resources necessary for training. Majority of the above studies train XGBoost surrogate models on thousands or millions of available input-output data pairs. The first goal of this paper, on the other hand, is to test whether this precedence of XGBoost over ANNs remains valid in situations when one has access to only tens or hundreds of simulation results, which would most often be the case during surrogate optimisation, whose basic underlying assumption is that it takes a long time to obtain a new datapoint.

Similarly, the above studies confirm that LHS is the most popular sampling method in building energy community. A sampling method should ideally be space-filling, so that sampled data points are evenly spread over the design space, and non-collapsing, so that when sampled points are projected along any coordinate, the minimum distance between their projections is as large as possible. LHS automatically produces non-collapsing samples, further guaranteeing space-filling when sample is optimised with respect to one of several distance-based criteria. However, LHS is a one-shot method whose sample size has to be known in advance, as it is not possible to extend it with a new sample point once it is generated.

A notable, but overlooked alternative to LHS is a Monte Carlo-based sampling method mc-intersite-proj-th (MIPT) [17], which produces samples iteratively by adding in each step an appropriately selected point to the current sample. If the current sample has *n* points, the (*n* + 1)-st sample point is obtained by taking *kn* random candidate points for some fixed *k* (say *k* = 100), discarding those with low projected distance from the current sample, and selecting among the remaining candidates the one with the maximum intersite distance from the current sample. Due to this, MIPT samples tend to have better space-filling and non-collapsing properties than LHS samples [17]. The second goal of this paper is to test whether this expected improvement of sample properties also leads to improvements in prediction quality of ANN and XGBoost surrogate models.

Precedence of XGBoost over ANN and of MIPT over LHS for surrogate optimisation studies is tested here on an engineering case study using which we have prepared a large set of synthetic data. Namely, in cases when one really has access to limited amount of data, it is usual to estimate the performance of ML models by dividing the available data into training, test and validation sets and then measure the loss metrics on test and validation sets during training. Such approach then suggests how much the trained ML models *should* be generalisable to the remaining unknown data. However, our goal here is to get an understanding of true generalisability of trained ML models and better judge the quality of surrogate models. From this reason, we have run simulations of the case study model for parameter combinations spread uniformly throughout its design space to create a large set of simulation results against which we compare predictions of ML models trained on very small subsets of this data. While it is customary in surrogate optimisation studies to use various mathematical test functions (see, e.g., [18]) to quickly and easily generate synthetic data, most such functions have extremal properties that are not necessarily found in real engineering problems. Additionally, we wanted our engineering problem to have two main and essential input parameters, with the remaining parameters determining its subcases, in order to be able to visualise model predictions for specific subcases. Our previous experience in studies of architectural shading [19, 20] led us to devise a simple case study model of an office cell with a single window and a fixed overhang, with overhang depth and height as two main inputs and with climate type, presence of obstacles, orientation and setpoints as auxiliary inputs. We have simulated all 729,000 variants of this model with EnergyPlus to determine their heating (*H*), cooling (*C*) and lighting (*L*) loads and equivalent primary energy needs (*E*), against which results we have then tested generalisability of ANN and XGBoost models trained on small samples of this data.

## 2 Methods

### 2.1 The office cell building energy model

**Fig 1:**
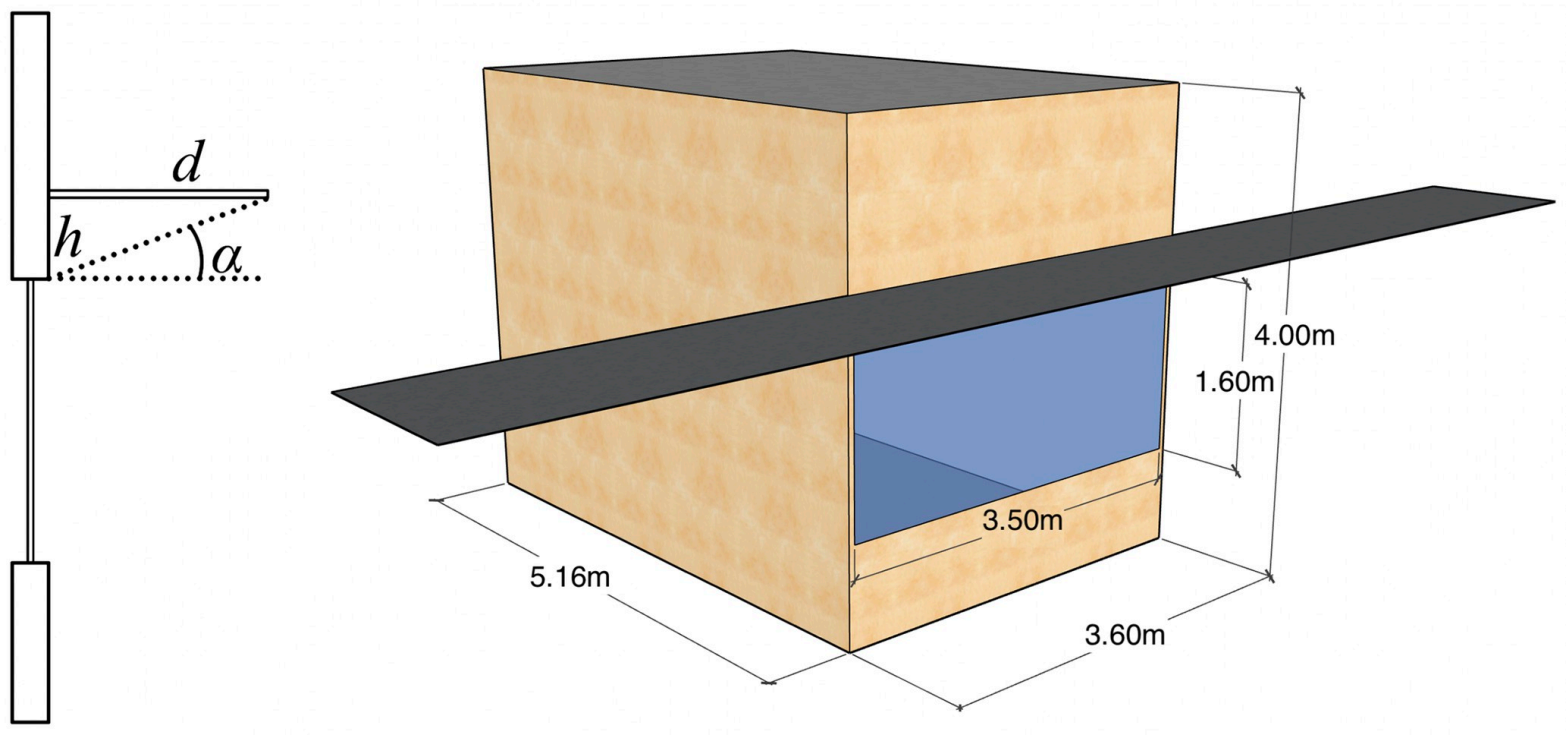
The office cell model used as the case study.

The case study is envisaged as a single-person office cell (see Fig 1) situated in the ASHRAE Standard 90.1-2019 PNNL large office building model [21], keeping intact most of its settings. The exterior wall construction is defined by the PNNL model, while the remaining walls are set as adiabatic. Additional data about the building energy model is summarised in Table 1.

**Table 1:** Properties of the office cell building model in various climates.

	Dubai	Honolulu	Tucson	San Diego	New York	Denver
Climate zone	extremely hot, dry (0B)	very hot, humid (1A)	hot, dry (2B)	warm, marine (3C)	mixed, humid (4A)	cool, dry (5B)
Exterior wall	20cm concrete wall, thermal insulation R = 0.85m^2^K/W, 13mm gypsum board, absorptances: solar = 0.7, visible = 0.5					
Interior wall	26mm gypsum board, absorptances: solar = 0.7, visible = 0.5					
Floor	10cm concrete floor, rough carpet pad, absorptances: solar = 0.7, visible = 0.8					
Ceiling	13mm acoustic ceiling, absorptances: solar = 0.7, visible = 0.2					
Glazing						
U-factor	2.84	2.84	2.61	2.38	2.04	2.04
Solar heat gain coefficient	0.23	0.23	0.25	0.25	0.36	0.38
Visible transmittance	0.25	0.25	0.275	0.275	0.396	0.418
Lighting power density	6.89W per m^2^ of floor area					
Electric equipment	8.075W per m^2^ of floor area					

EnergyPlus is set to recompute solar path, shadowing and diffuse sky modeling at each time step, to take into account shading by exterior surfaces and to project solar rays through window to compute the transmitted beam radiation reaching each surface in the zone. The daylight illuminance control linearly dims artificial lighting from 100% down to 20% or turns it off, using two daylighting sensors with the illuminance set point of 377 lux. Since the single office is considered here, its HVAC system is set to ideal loads.

Note that the choice of fixed overhang for the office cell was made primarily to facilitate comparisons in the present study. Extensive locations with cloudy winter climates benefit substantially from solar radiation scattered to the top of the sky dome by clouds. This beneficial radiation is blocked by fixed overhangs in winter, leading to prevalent use of operable shading for office windows in all but arid and semi-arid climates. Locations considered here include five sunny arid and semi-arid climates and only one cloudy humid temperate climate (NY).

The building model has several variable parameters whose possible values are listed in Table 2. These define a total of 6 × 81 × 25 × 5 × 3 × 2 × 2 = 729, 000 variants of the building model, whose heating, cooling and lighting loads are simulated by running EnergyPlus in parallel using eppy [22].

**Table 2:** The office cell building energy model parameter values.

Parameter	Possible values
Climate	0: Dubai
	1: Honolulu
	2: Tucson
	3: San Diego
	4: New York
	5: Denver
Overhang depth	from 0m to 1.60m in 0.02m steps
Overhang height	from 0.01m to 0.49m in 0.02m steps
Southern obstacle	0: none
	1: medium, 11am–2pm
	2: high, 11am–2pm
	3: medium, 3pm–5pm
	4: medium, 8am–10am and medium, 3pm–5pm
Office cell orientation	0: south
	-45: south-east
	45: south-west
Cooling set points	24°C or 26°C
Heating set points	19°C or 21°C

### 2.2 Training of machine learning models

Separate ANN and XGBoost models for heating, cooling and lighting loads, as well as equivalent primary energy needs, are trained for each building model variant from a small number *S* (initially *S* = 100) of sampled simulation results with surrogate model inputs based on the overhang depth *d* and height *h*. The samples of (*d*, *h*) pairs are chosen by maximin LHS from scikit-optimize [23] and two variants of MIPT [17], which slightly differ in the way the candidate points are randomly generated. Since the existing MIPT implementations are available for Matlab only [24–26], MIPT is reimplemented in Python for this study—it is accessible from mipt.py at [27].

One set of surrogate models was trained solely with *d* and *h* as inputs, while another set was trained with inputs expanded by the *α*-related values (see Fig 1): *h*/*d* (=tan *α*), *d*/*h* (=cot *α*), $h/\sqrt{d^{2}+h^{2}}$ (=sin *α*), $d/\sqrt{d^{2}+h^{2}}$ (=cos *α*), as well as the surface area *dh* and the diagonal length $\sqrt{d^{2}+h^{2}}$ of the rectangle below the overhang and above the window. Apart from tan *α* and cot *α*, all the inputs are normalised to the (0, 1) interval.

There is still no consensus in literature on the adequate ANN architecture for learning general unknown functions. Feed-forward ANNs with fully connected layers are used here, with ReLU: *f*(*x*) = max{0, *x*} as the activation function of neurons in hidden layers. If *a*_0_, …, *a*_*n*_ denote the numbers of neurons in the ANN layers (with *a*_0_ being the number of inputs and *a*_*n*_ being the number of outputs), then ANN has a total $\sum_{i=1}^{n}a_{i-1}a_{i}+\sum_{i=1}^{n}a_{i}$ trainable parameters, accounting for link weights and neuron biases. The number of trainable parameters of ANN has to be sufficiently smaller than the size of available training data (at most 100 here), in order to reduce the freedom of ANN to overfit. Since each ANN has 8 inputs and 1 output (prediction of either *H*, *C*, *L* or *E*), simple computation leads to relatively small maximum numbers of neurons in hidden layers for ANNs with 1–3 hidden layers that were chosen for this study (see Table 3). Each ANN uses Adam optimiser with the learning rate 0.003, as well as dropout layers with probability of 0.3, 1D batch normalisation layers and early stopping after 10 epochs without improvements to reduce overfitting. The ANNs are trained with pytorch [28], with training code given in train_ann.py at [27].

**Table 3:** Surrogate models used in the study.

Name	Description
N1	ANN(8,8,1) with ReLU activation, learning rate 0.003, 30% dropout and early stopping after 10 epochs
N2	ANN(8,5,5,1) with ReLU activation, learning rate 0.003, 30% dropout and early stopping after 10 epochs
N3	ANN(8,4,4,4,1) with ReLU activation, learning rate 0.003, 30% dropout and early stopping after 10 epochs
X1	XGBoost with learning rate 0.3 and early stopping after 10 rounds
X2	XGBoost with learning rate 0.1 and early stopping after 10 rounds
X3	XGBoost with learning rate 0.03 and early stopping after 10 rounds

XGBoost models in this study mostly use default hyperparameter values. They differ in learning rates which range from 0.03 to 0.3 (see Table 3)—smaller learning rates enable XGBoost to fit better to the training set, but also lead to larger number of regression trees in the trained ensemble. Early stopping after 10 rounds without improvement is employed for overfitting reduction. The XGBoost training code is found in train_xgb.py at [27].

ANN and XGBoost models are trained by sampling *S* pairs of (*d*, *h*) values from the space 0 ≤ *d* ≤ 2m, 0 ≤ *h* ≤ 0.5m, with the corresponding simulated values of *H*, *C*, *L* or *E* acting as the output values. To enable all sampled data points to impact both training and testing phases, 5-fold cross validation is used: the sample is randomly divided into 5 folds of *S*/5 data points each, and an ensemble of 5 ML models is then trained by taking in turn each fold as the test set and the remaining four folds as the training set. The final prediction of the surrogate model is the average of predictions of all five ML models in this ensemble. The loss function used for training both ANN and XGBoost models is the mean squared error

$$MSE=\frac{1}{n}\sum_{i=1}^{n}{\left(Y_{i}-{\hat{Y}}_{i}\right)}^{2},$$

between the vectors *Y* of simulated values and $\hat{Y}$ of ML model predictions.

ANN and XGBoost models are trained separately for each of 360 combinations of climate, obstacle type, orientation and heating and cooling set points. Since all 729,000 building model variants are already simulated, the prediction quality of surrogate models is measured by the coefficient of variation of the root mean squared error

$$CV\left(RMSE\right)=\frac{\sqrt{\frac{1}{n}\sum_{i}{\left(Y_{i}-{\hat{Y}}_{i}\right)}^{2}}}{\frac{1}{n}\sum_{i}Y_{i}},$$

for all 2,025 combinations of *d* ∈ {0, 0.02m, …, 1.6m} and *h* ∈ {0.01m, 0.03m, …, 0.49m}. Smaller *CV*(*RMSE*) thus represents a better surrogate model.

### 2.3 Automating surrogate model training

Python methods developed for this study are collected into the package overhang_surrogates [27], that can also be installed directly with pip. It allows interested researchers to produce samples with MIPT [17], train XGBoost surrogate models with *k*-fold cross validation for a given pandas dataframe, and produce 3D diagrams for selected pandas dataframe columns with vedo.

The method


MIPT(n, dim=2, alpha=0.5, k=100)


returns a sample of n points from the dim-dimensional hypercube [0, 1]^dim^. The values alpha and k influence the selection of new sample points: after *s* points have been selected into the sample, new k*s* candidate random points will be generated with projected distance at least alpha/*s* from the existing *s* sample points, after which the candidate point with the largest distance from the previous *s* points is selected as the (*s* + 1)-st sample point. An existing sample can be extended with


MIPT_extend(sample, n, alpha=0.5, k=100)


which adds n new points to sample according to the above requirements. Due to this iterative sampling strategy, if one wants to construct two samples with, say, 50 and 100 points, it is enough to construct the larger sample by calling MIPT(n = 100), and then directly take its first 50 points as the smaller sample. Auxiliary methods hypercube_to_indices and indices_to_hypercube convert the sample points from [0, 1]^*d*^ to the indices of *d* arrays of given lengths, and back.

Assuming the pandas dataframe df contains output values in the columns meter_cols, that should be predicted from the input values in columns input_cols (obtained, e.g., by using the method sample_and_simulate_overhangs), the method


train_model_ensembles(df, input_cols, meter_cols,
folds=5, learning_rate=0.1, early_stopping_rounds=10)


will train a separate ensemble of XGBoost models for each column in meter_cols, using cross validation with given number of folds, the learning_rate and the number of early_stopping_rounds. The method predict_meters can then be used to make predictions for arbitrary input values.

The method


make_3d_diagram(df, xcol, ycol, zcol,
  minxvalue=None, maxxvalue=None, numxticks=10,
  minyvalue=None, maxyvalue=None, numyticks=10,
  minzvalue=None, maxzvalue=None, numzticks=10,
  aspect_ratio=(1, 1, 1), camera=(-15, 25), ...)


produces a 3D diagram for data in the columns xcol, ycol and zcol of the dataframe df, assuming df contains a row for each pair in the Cartesian product of values in xcol and ycol, with the whole diagram shown in a grid box described by the remaining parameters.

With the help of these methods, data and diagrams shown in Figs 9 and 10 can be obtained basically as follows:


import overhang_surrogates as ovs
df_sampled = ovs.sample_and_simulate_overhangs(
             'm1NewYork.idf', 'm1NewYork.epw', 
             'Office_Cell_Wall_South_Window',
             np.linspace(0.0, 1.6, 81), 
             np.linspace(0.01, 0.49, 25), 10.5, 100)
models = ovs.train_model_ensembles(df_sampled, 
         ['depth', 'height'], 
         ['DistrictHeating:Facility', 
          'DistrictCooling:Facility',
          'InteriorLights:Electricity']
df_predicted = ovs.predict_meters(models, 
          {'depth': np.linspace(0.0, 1.6, 81),
           'height': np.linspace(0.01, 0.49, 25)}          
ovs.make_3d_diagram(df_predicted, 'depth', 'height', 
                   'DistrictHeating:Facility')
ovs.make_3d_diagram(df_predicted, 'depth', 'height', 
                   'DistrictCooling:Facility')
ovs.make_3d_diagram(df_predicted, 'depth', 'height', 
                   'InteriorLights:Electricity')


## 3 Results and discussion

**Fig 2:**
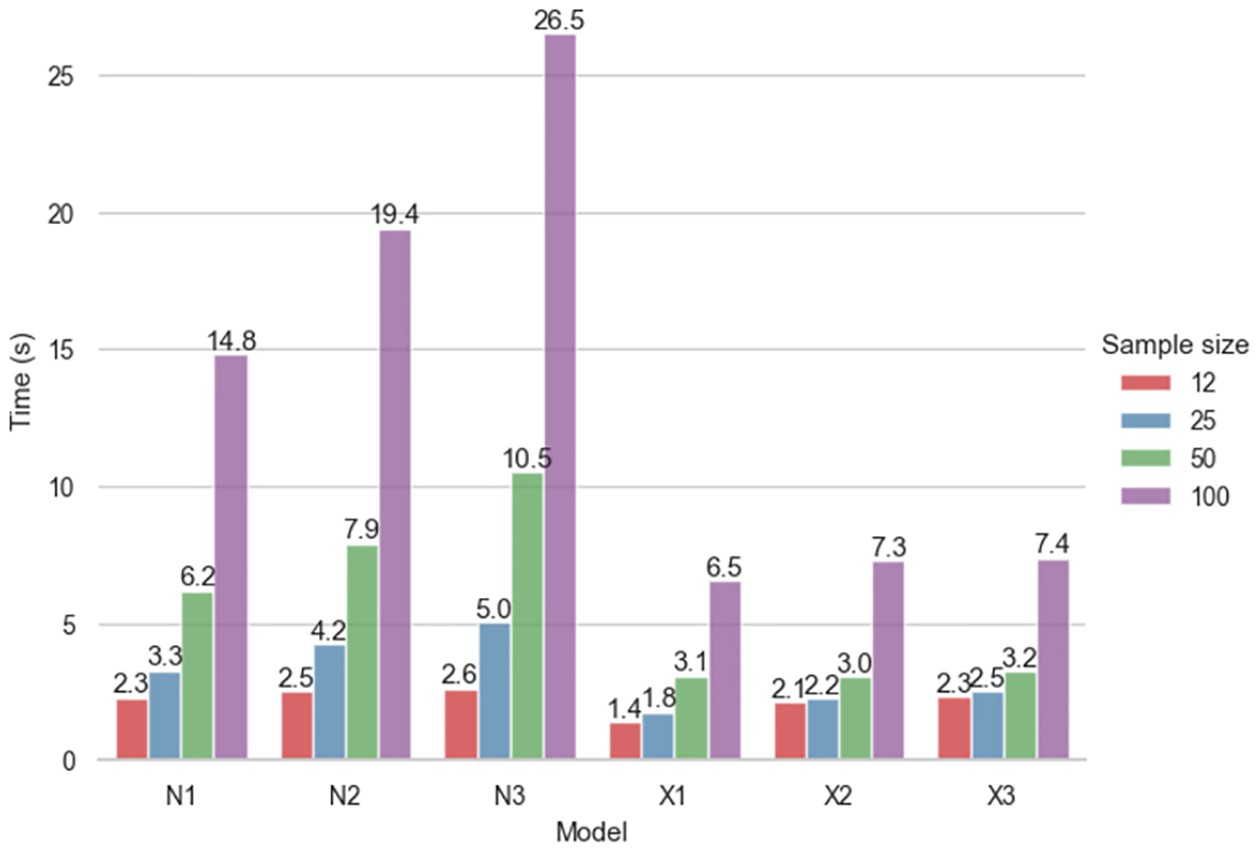
Average time needed to train various ML models using 5-fold cross validation for all four simulated values (*H*, *C*, *L* and *E*) for one combination of climate, obstacle type, orientation and heating and cooling set points of the building model, depending on the sample size. The ML models N1, N2 and N3 are ANNs with one, two and three hidden layers of neurons, respectively, while X1, X2 and X3 are XGBoost models with learning rates set at 0.3, 0.1 and 0.03, respectively.

EnergyPlus simulations of the 729,000 variants of the office cell building energy model were run in parallel on four Ubuntu Linux PCs with 8-core i7 processors for approximately one week (simulation results are available online at [29]). Separate ML model ensembles were further trained for each of the 8,640 combinations of the following office cell parameters: climate, presence of obstacles, orientation, heating and cooling set points, as well as the number of input columns, sampling method and load type. Training three XGBoost models for these 8,640 combinations lasted about two days in total on a MacBook Air laptop with the 8-core M1 processor. In comparison, training three ANN models (with 1, 2 or 3 hidden layers) using pytorch for these 8,640 combinations lasted about seven days in total on the same computer. Fig 2 shows the average time needed to train these ML models using 5-fold cross validation, depending on the sample size, for one combination of parameters of the building model. As one can see from this figure, training different XGBoost models for a given sample size takes relatively similar time regardless of the learning rate, while time needed to train ANN models strongly depends on the number of hidden layers.

This section presents estimates of the prediction quality of obtained surrogate models, and discusses the impact of the number of inputs, the sampling method, the ML model architecture, the office cell parameters, and the sample size on the surrogate prediction quality.

### 3.1 Impact of the number of inputs

Each ML model for each building model variant was trained separately for the case of two inputs normalised to the interval [0, 1]: *d*/2 and 2*h*, and for the case of eight inputs:*d*/2, 2*h* and their derived functions $h/\sqrt{d^{2}+h^{2}}=\text{sin}\;\alpha$, $d/\sqrt{d^{2}+h^{2}}=\text{cos}\;\alpha$, $\sqrt{(d^{2}+h^{2})/4.25}$, *dh*,*h*/*d* = tan *α* and *d*/*h* = cot *α*, among which the first six are normalised to [0, 1]. The same model architecture was used for both cases of two and eight numerical inputs, so that in the case of two inputs, the additional numerical inputs expected by the ML models were filled with constant zero values.

**Fig 3:**
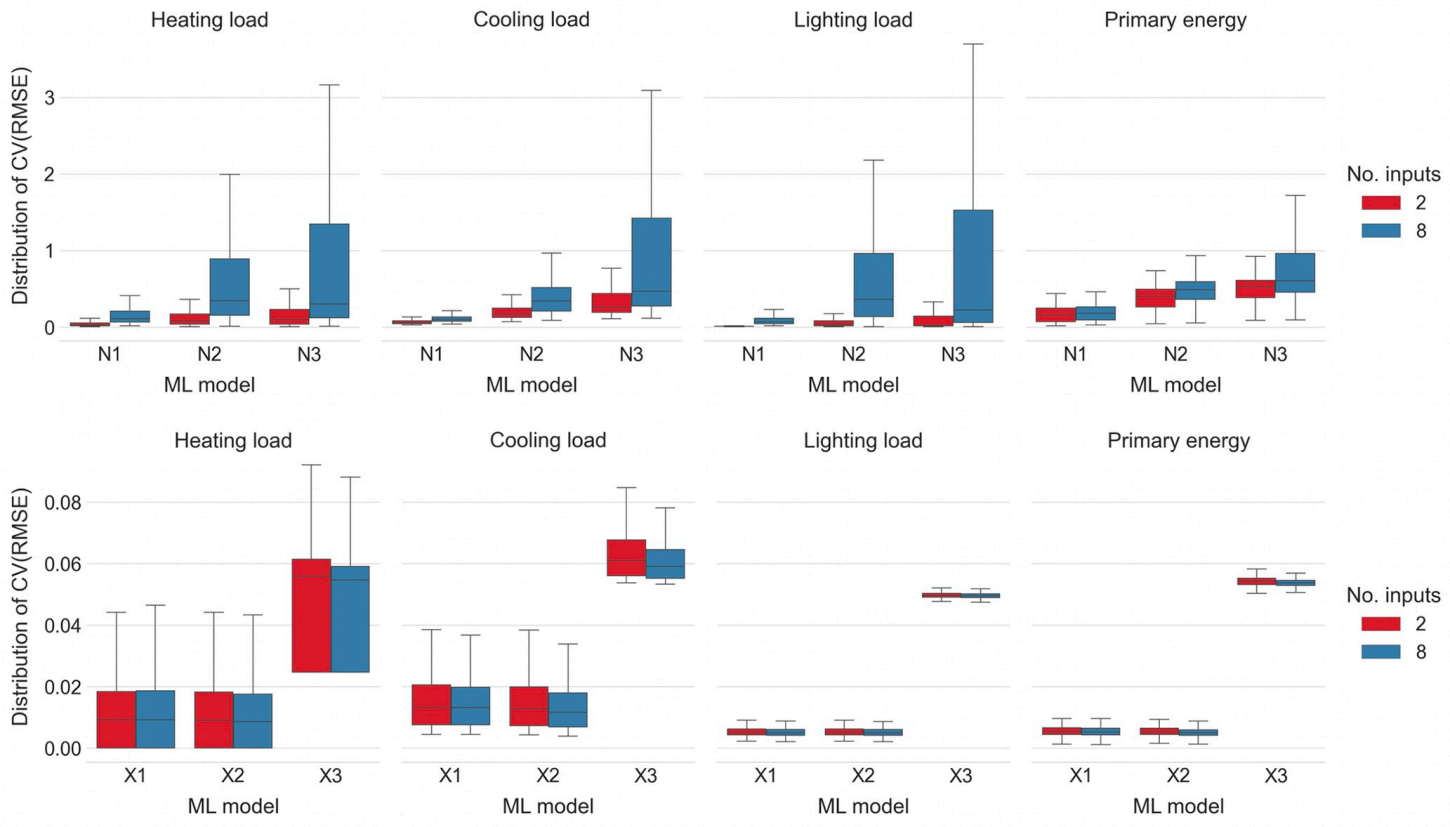
Distribution of *CV*(*RMSE*) values for the cases of 2 inputs and 8 inputs over different ML models and different loads. The ML models N1, N2 and N3 are ANNs with one, two and three hidden layers of neurons, respectively, while X1, X2 and X3 are XGBoost models with learning rates set at 0.3, 0.1 and 0.03, respectively.

Fig 3 shows that the presence of additional varying numerical inputs, in a sense, “confuses” ANN-based models which return significantly higher *CV*(*RMSE*) values than their counterparts which use only *d*/2 and 2*h* as inputs. Possible explanation for this behaviour may be that ANN-based models with larger number of varying numerical inputs need to be trained for a larger number of epochs to reach appropriate levels of dependence of their predictions on the input values. Since the overarching goal is to create acceptable surrogate models for *H*, *C*, *L* and *E* from a relatively small sample size and in relatively short time, the case of ANN-based models with eight inputs was discarded in the sequel.

On the other hand, one can also see from Fig 3 that additional numerical inputs do slightly decrease the average *CV*(*RMSE*) values of the XGBoost-based models. This improvement in the average *CV*(*RMSE*) values is on average 2.63% across all XGBoost-based models and all loads, with the only increase of 0.05% in the average *CV*(*RMSE*) values present while predicting heating loads with the XGBoost model with the learning rate 0.3. Thus in the sequel only XGBoost models trained with all eight numerical inputs are considered.

### 3.2 Impact of the sampling method

**Fig 4:**
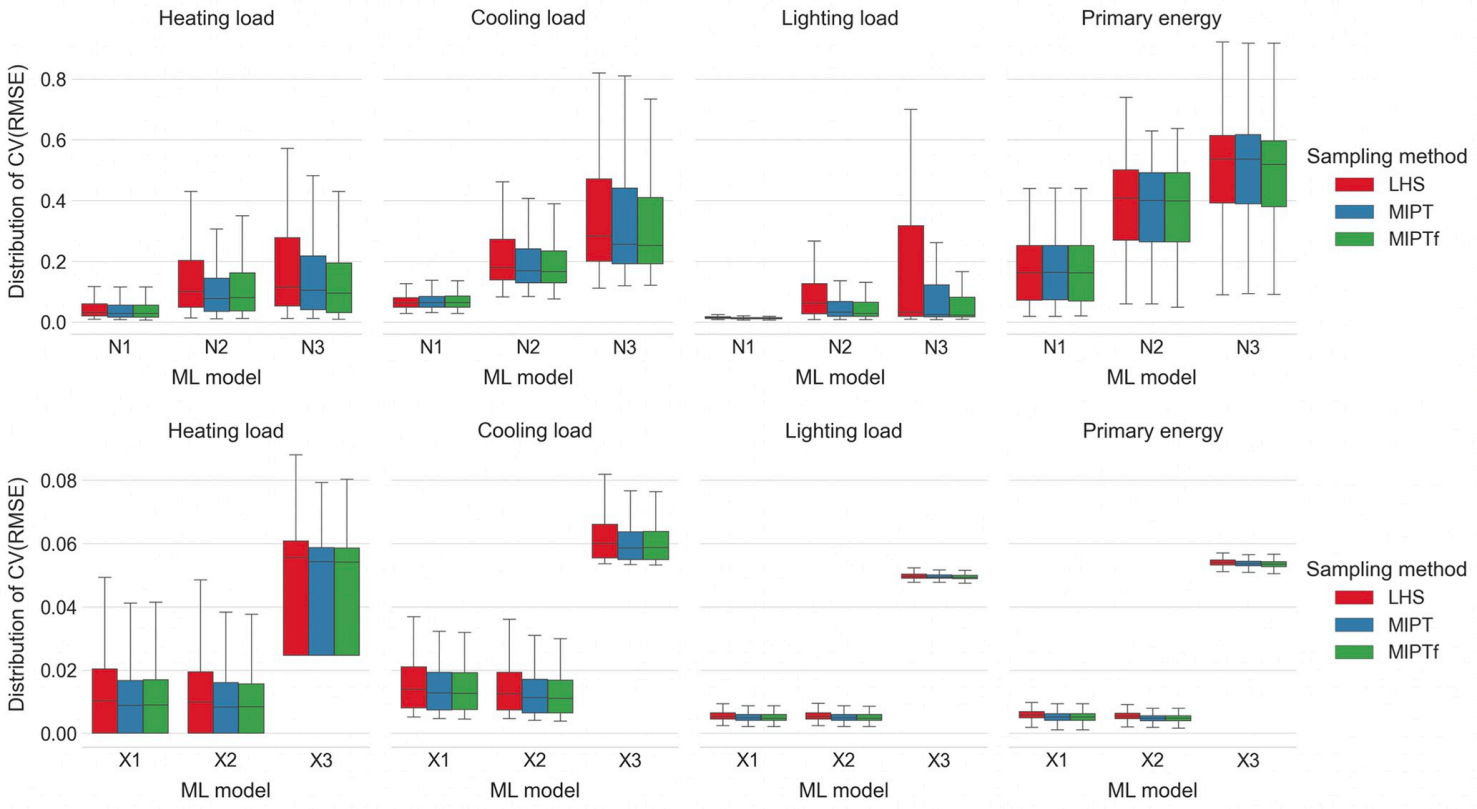
Distribution of *CV*(*RMSE*) values for the cases of LHS, MIPT and MIPT_f_ sampling methods over different ML models and different loads. The ML models N1, N2 and N3 are ANNs with one, two and three hidden layers of neurons, respectively, while X1, X2 and X3 are XGBoost models with learning rates set at 0.3, 0.1 and 0.03, respectively.*CV*(*RMSE*) values were computed for the ANN models with two inputs and the XGBoost models with eight inputs, as suggested in Subsection 3.1.

Each ML model was also trained separately with three sampling methods: LHS, MIPT and MIPT with avoidance of forbidden hypercubes (MIPT_f_, see [17]). LHS is one-shot method that simultaneously selects all sample points, while MIPT and MIPT_f_ are iterative methods that select a new sample point with respect to the previously selected sample points. As a result, the samples produced by MIPT and MIPT_f_ tend to have larger minimum intersite and minimum projected distance between the sample points than the samples produced by LHS, albeit at the expense of a somewhat longer running time: approximately 1.1s for 100 sample points produced by LHS compared to 10.2s for 100 sample points produced by either MIPT or MIPT_f_ on a MacBook Air laptop with the M1 processor. Fig 4 confirms that larger minimum intersite and minimum projected distance between sample points offered by MIPT and MIPT_f_ sampling methods, which translates to more evenly spread sample points over the input domain, has noticeably positive effects on the prediction capabilities of almost all considered ML models. The differences between MIPT and MIPT_f_ sampling methods are negligible in all three aspects: in the running time, in the quality of samples and in the impact on prediction capabilities of ML models (with MIPT_f_ leading to slightly better prediction capabilities). Theoretically, it is possible for MIPT to discard all candidate points from one iteration, provided they all happen to be too close in projected distance to the already sampled points. On the other hand, MIPT_f_ avoids this (highly unlikely) possibility by specifically choosing candidate points from regions with sufficiently large projected distance from already sampled points, and thus chooses a new sample point in each iteration, which is why MIPT_f_ is used in the sequel.

### 3.3 Impact of the ML model architecture

**Fig 5:**
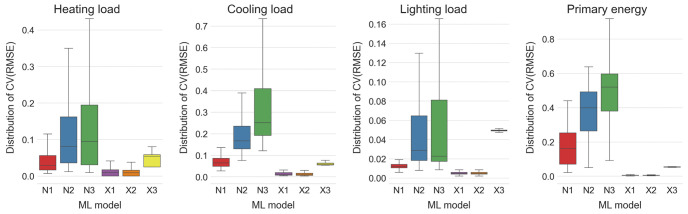
Distribution of *CV*(*RMSE*) values for different ML models and different loads. The ML models N1, N2 and N3 are ANNs with one, two and three hidden layers of neurons, respectively, while X1, X2 and X3 are XGBoost models with learning rates set at 0.3, 0.1 and 0.03, respectively. The axes for *CV*(*RMSE*) values were set individually due to differences in their ranges for different loads. Outliers, which are especially present for models N2 and N3, are not shown. Following suggestions in Subsections 3.1 and 3.2, the coefficients of variation were computed for the sample points selected by the MIPT_f_ sampling method, the ANN models with two inputs and the XGBoost models with eight inputs.

Fig 5 shows the box plots of the distributions of *CV*(*RMSE*) values for the ANN models with two inputs and the XGBoost models with eight inputs, trained on the samples selected by MIPT_f_. From the very high variability of *CV*(*RMSE*) values for models N2 and N3 (and a large number of their outliers which are not shown in Fig 5), one can immediately see that ANNs with several hidden layers of neurons require far longer training time to reach convergence than what is allowed by the early stopping criterion here. In such restricted training time setting, the model N1 with a single hidden layer achieves far better prediction capabilities than its deeper counterparts N2 and N3.

On the other hand, Fig 5 clearly indicates that the XGBoost models with the appropriately selected learning rates significantly outperform the ANN models in this limited setting: for all loads, the upper quartile of X2 is positioned lower than the lower quartile of N1. Similar ascendancy was also observed in the ASHRAE Great Energy Predictor III competition [9], which showcased the predominance of gradient-boosted tree models in predicting whole building energy consumption. In addition, the XGBoost models sport more uniformly distributed *CV*(*RMSE*) values with significantly narrower inter-quartile ranges than their ANN counterparts. Fig 5 also shows the importance of properly setting the learning rate for the XGBoost models: while the increase of the learning rate from 0.1 to 0.3 only slightly decreases the predictive capabilities of X1 model, the decrease of the learning rate from 0.1 to 0.03 significantly worsens the predictive capabilities of X3 model. Since the best learning rate for XGBoost models in this case study appears to be situated in the vicinity of 0.1, X2 is selected as the ML model of choice in the sequel.

### 3.4 Impact of the office cell model parameters

**Fig 6:**
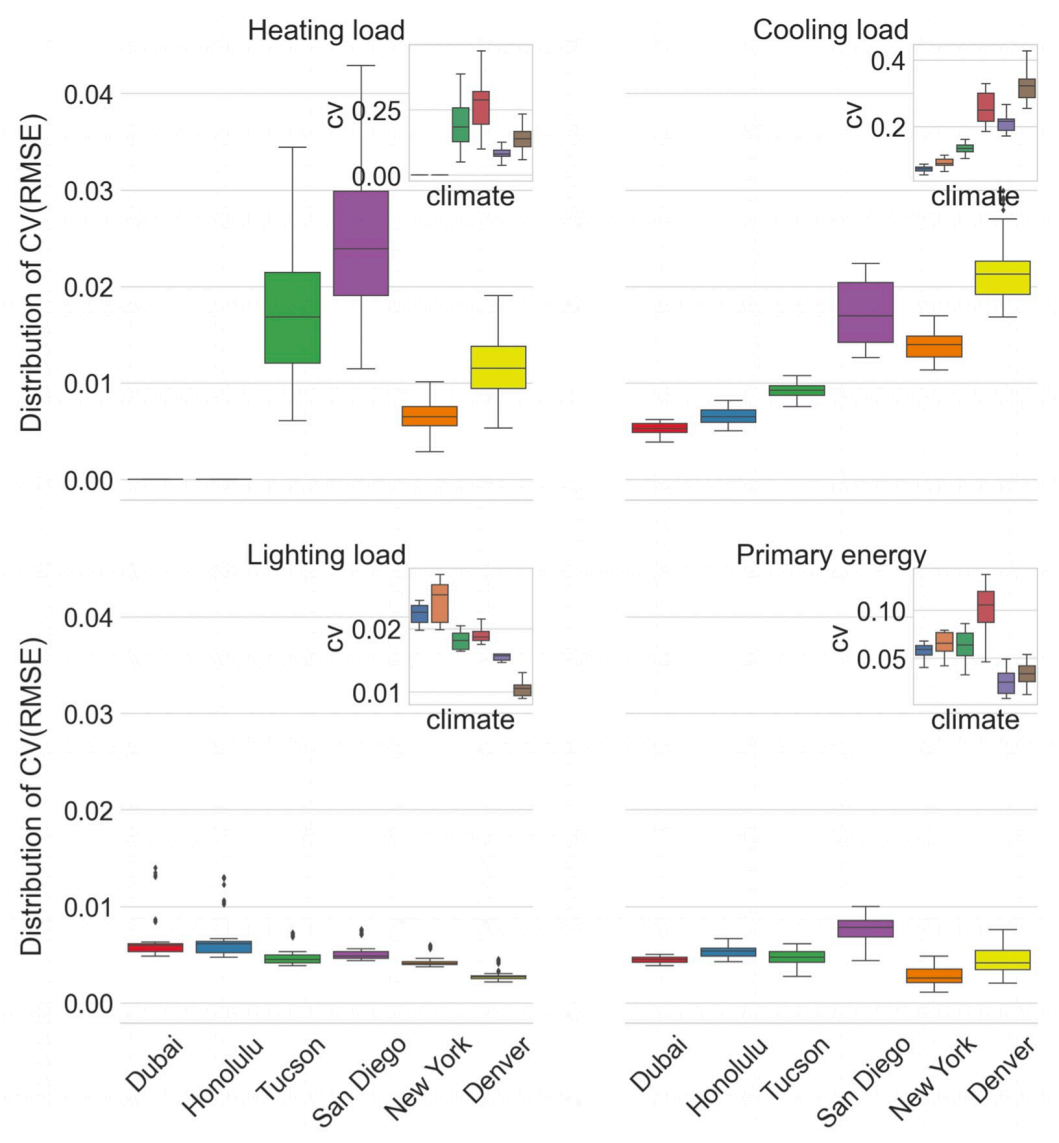
Distribution of *CV*(*RMSE*) values for the best performing XGBoost models X2 with learning rate 0.1, eight inputs and MIPT_f_ sampling for different loads in different climates. Insets shows the distributions of CV values of the simulated loads for the office cell model in these climates.

Fig 6 shows the distribution of *CV*(*RMSE*) values for the best performing XGBoost models X2 with learning rate 0.1, eight inputs and MIPT_f_ sampling) for different loads in different climates. These models turn out to be most certain when predicting lighting loads and equivalent primary energy needs, while their predictions of cooling and, especially, heating loads have much higher variability of root mean squared error (RMSE). This becomes even more interesting when one takes into account that the equivalent primary energy need is actually a linear combination of these loads.

A general observation is that the model X2 tends to achieve better predictive capabilities when the simulated loads for the whole population are less dispersed (and thus, with high probability, also the loads of the sampled points on which the model is trained). This may be visually inspected in Fig 6, where next to the distribution of *CV*(*RMSE*) values each inset, for each load type in each climate, further shows the distribution of the coefficient of variation (*CV*, the ratio of the standard deviation to the mean value) of the loads simulated by EnergyPlus. These insets reveal a strong visual resemblance between the dispersion of the root mean squared error between the predicted and simulated loads and the dispersion of the simulated loads per se. This resemblance also appears when other office cell model parameters are varied, apart from the building model orientation.

A probable explanation for this resemblance may lie in the structure of XGBoost ensemble models. They consist of a collection of regression trees, each of which uses decision rules on the components of the input data to assign a constant score from some of its leaves to an instance of the input, with the final prediction being the sum of scores assigned from each regression tree. Hence the prediction function of an XGBoost model is a sum of piecewise constant functions, which in itself is a piecewise constant function on the input domain partitioned by the conjuction of the decision rules used in the constituent regression trees. Thus, if the values to be predicted have smaller dispersion or, which is more-or-less equivalent, have smaller gradients, then it could be expected that the smaller number of piecewise constant functions may be sufficient for a good prediction. Put in another way, the same number of regression trees in an XGBoost model could be expected to yield more precise predictions for data that is less dispersed and has smaller gradients.

Heating and cooling loads for the office cell model in this study, have almost constantly slanted shapes, with gradients bounded away from zero, when considered as functions of *d* and *h*. On the other hand, the diagrams of equivalent primary energy needs have larger nearly horizontal areas with much smaller gradients (an example of the diagrams of these loads is shown in Figs 9 and 10). This apparently makes it easier for the X2 models to better predict the equivalent primary energy needs as piecewise constant functions, than the heating and cooling loads as its constituent terms.

Note that the above discussion does not refer to the actual magnitude of the loads, but only to their relative dispersion with respect to the mean value. Heating loads in New York (mean = 48.51kWh/m^2^) and Denver (mean = 36.61kWh/m^2^) are definitely higher than in San Diego (mean = 1.59kWh/m^2^) and Tucson (mean = 2.99kWh/m^2^), but they are far less dispersed (with CVs equal to 0.21, 0.26, 0.97 and 0.63 in New York, Denver, San Diego and Tucson, respectively), implying more precise XGBoost predictions of heating loads for New York and Denver than for San Diego and Tucson. The X2 models similarly achieve better prediction performance for the climates of Dubai, Honolulu and Tucson, which have larger, but less dispersed cooling loads than for the climates of San Diego, New York and Denver, whose cooling loads are much smaller, but more dispersed.

### 3.5 Impact of the sample size

**Fig 7:**
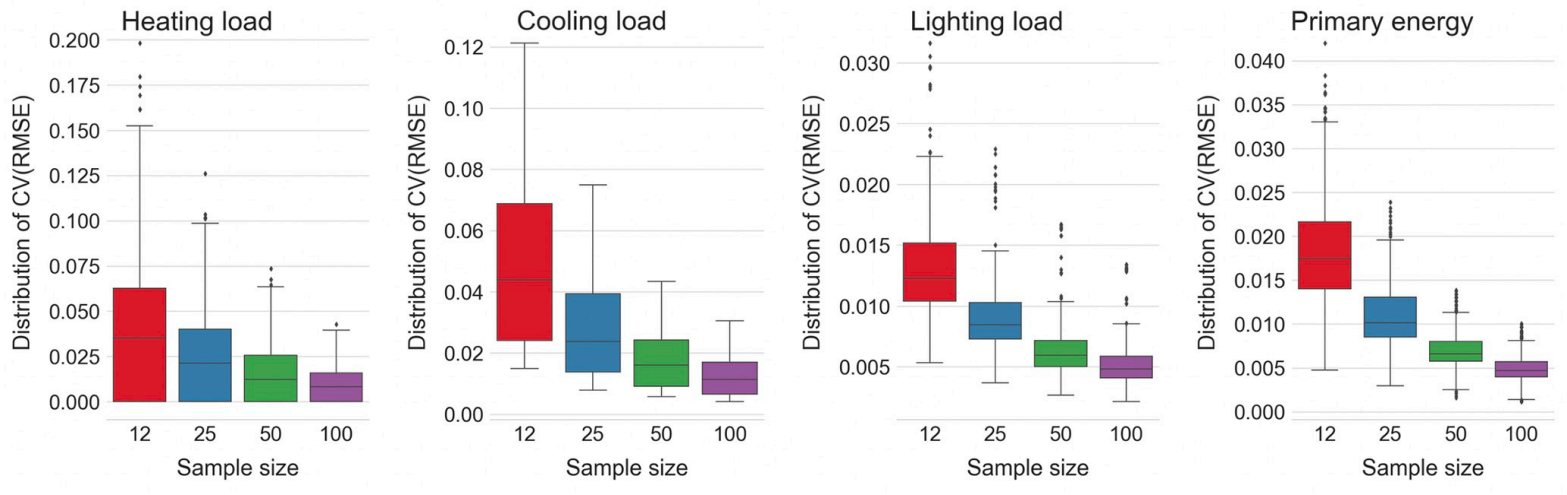
Distribution of *CV*(*RMSE*) values for the best performing X2 models with learning rate 0.1, eight inputs and MIPT_f_ sampling, trained over the samples with varying sizes containing 12, 25, 50 and 100 points, respectively.

Fig 7 shows distributions of *CV*(*RMSE*) values for the predictions of heating, cooling and lighting loads and equivalent primary energy needs of the four XGBoost models with learning rate 0.1, eight inputs and MIPT_f_ sampling, but this time trained on samples of different sizes: with either 12, 25, 50 or 100 (*d*, *h*) pairs out of 2,025 (*d*, *h*) combinations for each building model variant. These XGBoost models will be aptly named X12, X25, X50 and X100 in this subsection.

It can be immediately noticed that *CV*(*RMSE*) values of these surrogate models tend to decrease exponentially with the doubling of the sample size. Results of building energy simulations are valuable in decision making even though they do not always replicate the actual performance of built products. Actually, results of EnergyPlus simulations for shading and interior solar distribution may deviate by up to 4.44% from the analytically obtained results [30, Sections 2.8 & 2.10]. Fig 7 shows that *CV*(*RMSE*) values for heating and cooling loads fall within this range already for X50 (apart from a handful of outliers for heating load), and for lighting loads and equivalent primary energy needs already for X25 and even X12. This suggest that, to obtain useful surrogate models for the actual problem at hand, one should first train XGBoost model ensemble on a sample of smaller size and check its *CV*(*RMSE*) performance on the test set, before deciding whether to continue training with the doubled sample size.

### 3.6 Visualising actual predictions

**Fig 8:**
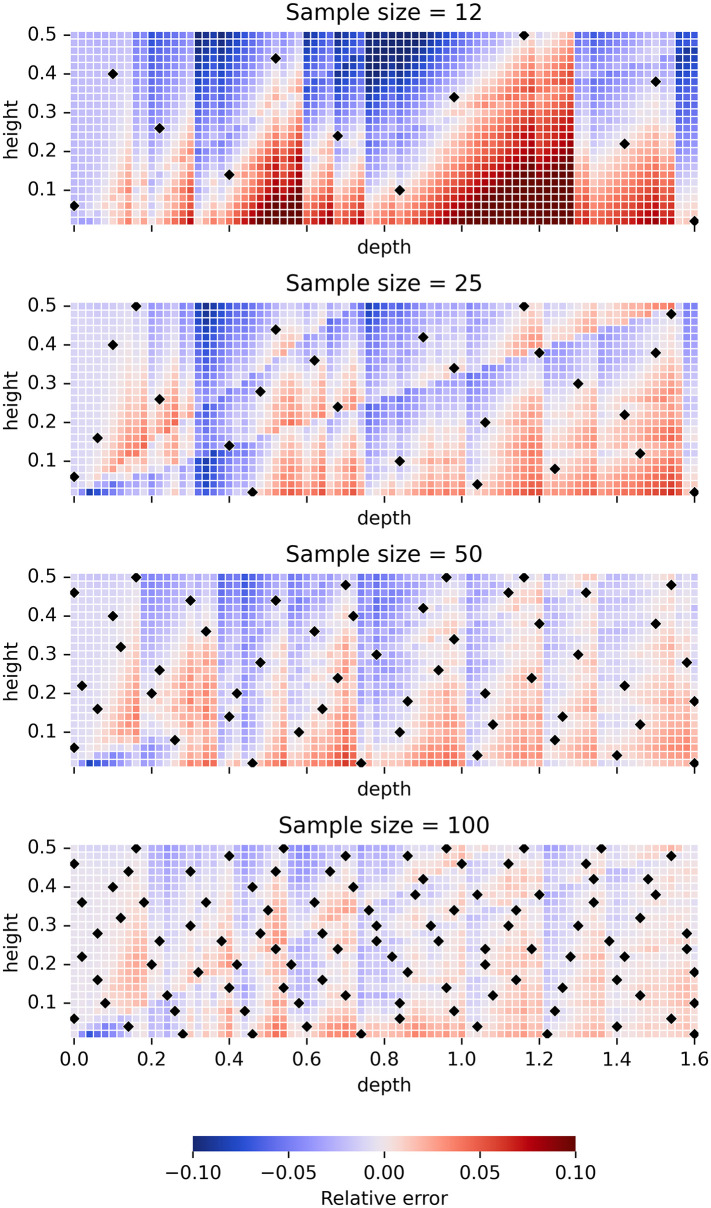
Relative errors of cooling load predictions for the four X2 models with learning rate 0.1, eight inputs and MIPT_f_ sampling, trained over the samples of sizes 12, 25, 50 and 100, respectively, for the particular case of New York climate with southern orientation, no obstacles, *hsp* = 21°C and *csp* = 24°C. Black diamond shapes depict the sample (*d*, *h*) points whose simulated loads were used for training the models. The samples were built iteratively, so that the sample of size 12 represents the first 12 points of the largest sample of size 100, etc.

**Fig 9:**
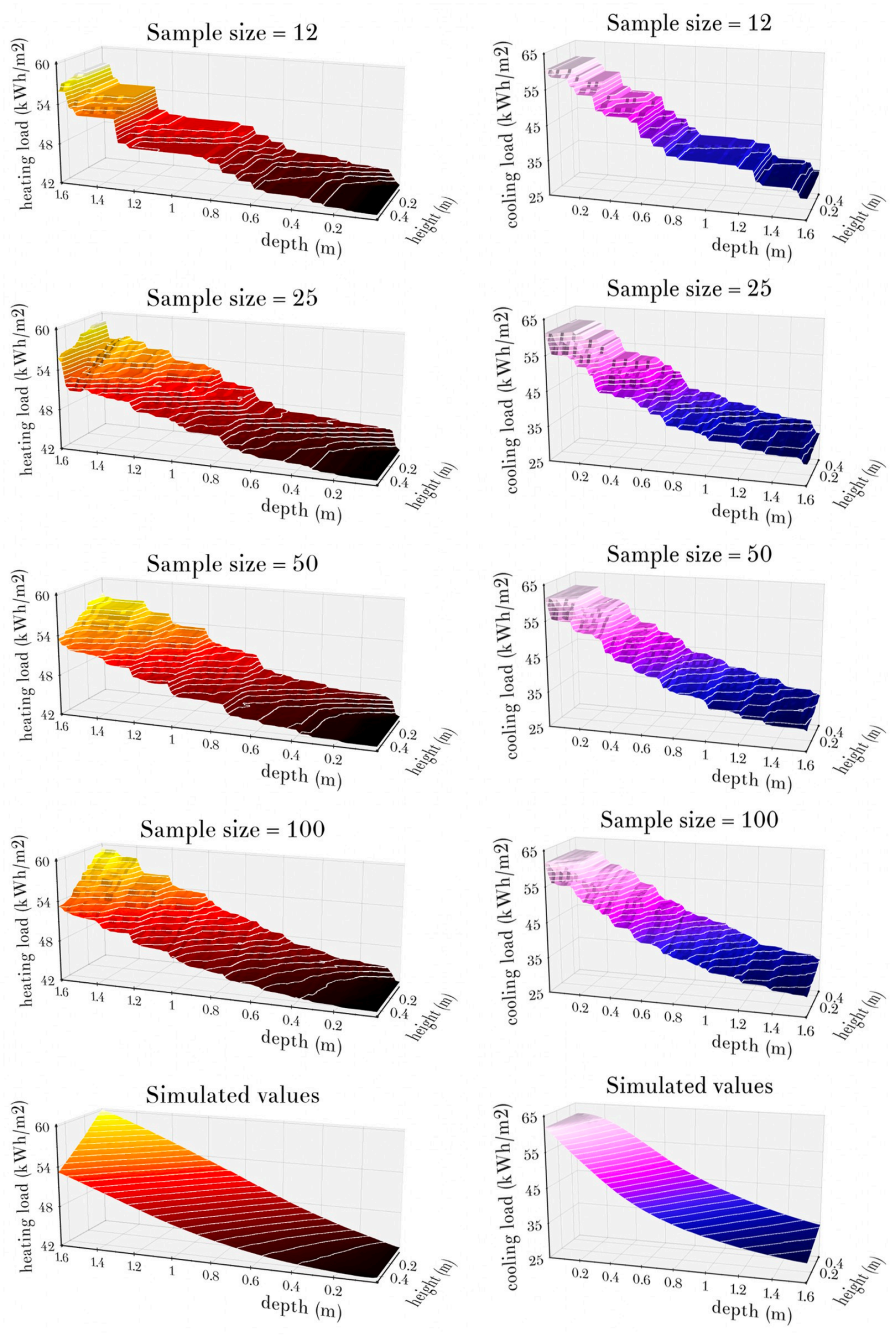
Diagrams of the predictions of heating loads (left column) and cooling loads (right column) of the XGBoost model ensembles X12, X25, X50 and X100, together with the actual simulated loads shown in the last row, for the office cell model in the New York climate with southern orientation, no obstacles, *hsp* = 21°C and *csp* = 24°C.

**Fig 10:**
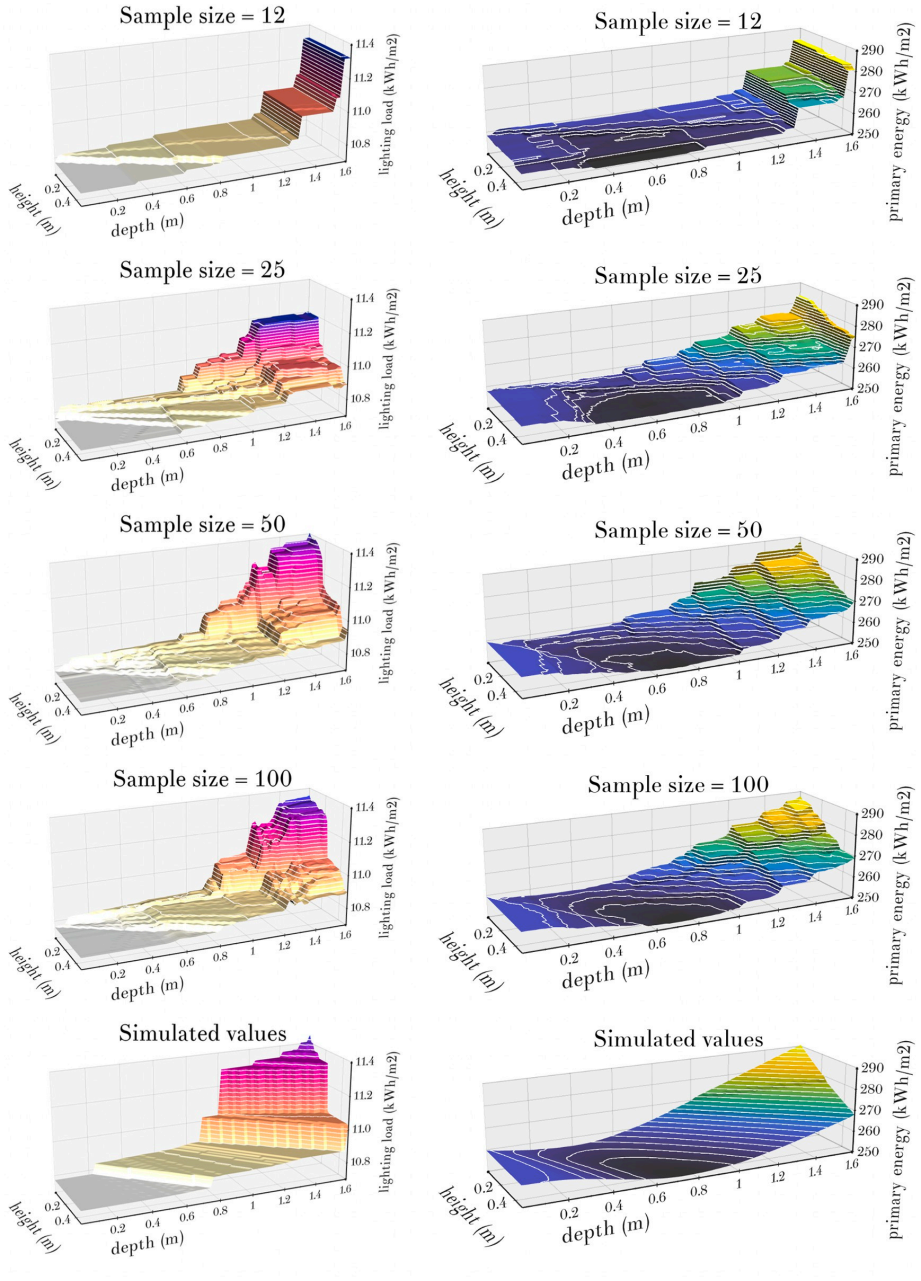
Diagrams of the predictions of lighting loads (left column) and equivalent primary energy needs (right column) of the XGBoost model ensembles X12, X25, X50 and X100, together with the actual simulated loads shown in the last row, for the office cell model in the New York climate with southern orientation, no obstacles, *hsp* = 21°C and *csp* = 24°C.

Since *CV*(*RMSE*) is a global indicator that summarises quality of model predictions over the larger set of 360 office cell model variants (with different climates, types of obstacles, orientations, and heating and cooling set points), the actual predictions of X12, X25, X50 and X100 models for a particular case of the New York climate with southern orientation, no obstacles, *hsp* = 21°C and *csp* = 24°C are illustrated in Figs 8–10.

Fig 8 clearly indicates the evolution of relative errors of cooling load predictions for these four models when the sample size is doubled. Important characteristic of the diagrams in this figure is their division into smaller parts using a collection of vertical and diagonal (and occasionally horizontal) lines that serve as their boundaries, in such a way that the relative error is continuously changing within these parts, but experiencing smaller or larger jumps when crossing the boundary between two parts. This division is an artefact of the already mentioned structure of XGBoost model ensembles that constrains their predictions to be piecewise constant functions. From the boundary lines observable in the diagrams of Fig 8 it can be seen that the most important input features used in the decision rules turn out to be the overhang depth *d* (leading to vertical lines), the ratio *d*/*h* (leading to diagonal lines of varying slopes) and the overhang height *h* (leading to horizontal lines). Analogous boundary lines appear in the diagrams of relative errors of predictions for *H*, *L* and *E*, which is why we skip them here.

The fact that the predictions of XGBoost models are piecewise constant functions is easily observable from the visualisations of actual predictions of the models X12, X25, X50 and X100 for heating and cooling loads in Fig 9, and lighting loads and equivalent primary energy needs in Fig 10. These diagrams also illustrate how the increase in the sample size makes it possible to introduce additional decision rules into regression trees, which in turn decreases areas of individual horizontal parts of prediction functions and leads to better fit between model predictions and actual loads. Although increased sample size quickly leads to more than acceptable levels of discrepancy between model predictions and actual loads, it is apparent from these diagrams that the piecewise constant XGBoost model predictions can hardly evoke smoothness of the shapes of heating and cooling loads and equivalent primary energy needs that are shown in these diagrams as well. Moreover, comparing the sequence of predictions of lighting loads from Fig 10 with the simulated lighting loads, it can be seen that these XGBoost models, despite their increasing accuracy, fail to converge to the shape of simulated lighting loads, which is probably the consequence of the fact that their constituent regression trees mostly use decision rules based on *d*, *h* or *d*/*h* (with latter leading to “rays” emanating from the point (*d*, *h*) = (0, 0) in Fig 10), while the “waterfall” shape of lighting loads follows straight lines determined by the ratios of *d* and *h* which are shifted by different amounts each.

## 4 Conclusions

This study compared the quality of predictions of ANN and XGBoost surrogate models for heating, cooling and lighting loads and equivalent primary energy needs, trained on relatively small number of results of EnergyPlus simulations for different variants of the office cell model that was used as a case study.

The immediate outcome is that XGBoost models provided more precise predictions for these loads. It was further observed that the use of Monte Carlo-based mc-intersite-proj-th [17] method for sampling, instead of the maximin Latin hypercube sampling, led to slightly improved quality of model predictions for both ANN and XGBoost models.

Being essentially collections of piecewise constant functions, XGBoost models tend to achieve better predictive capabilities when the simulated loads are less dispersed or have smaller gradients on the greater part of the design space. In such cases it could be expected that a smaller number of regression trees would be sufficient for a good prediction, or alternatively, that the same number of regression trees in an XGBoost model would yield more precise predictions. Consequently, XGBoost models for the present case study are more precise for the lighting loads and the equivalent primary energy needs than for the heating and the cooling loads, whose simpler slanted shapes have higher gradients bounded away from zero.

XGBoost models here were trained on samples with exponentially increasing sizes: 12, 25, 50 and 100, and it was observed that *CV*(*RMSE*) values of their predictions tend to decrease exponentially with a doubling of the sample size. This suggests that, in general, a binary search procedure could be used in training of XGBoost surrogate models: first train on smaller samples, and then double the sample size and retrain until satisfactory prediction quality is attained. For the present case study, taking into account inherent inaccuracies of EnergyPlus simulations, surrogate models of acceptable quality were obtained with samples of size 50 for heating and cooling loads, while samples of size 25 were sufficient for lighting loads and equivalent primary energy needs.

To conclude, XGBoost has already started to take hold in BPS community, as mentioned in the references, mostly in the domain of prediction models trained on large amounts of measured data. This study points out that, in combination with MIPT sampling, XGBoost has a substantive potential to yield good quality surrogate models also in the cases when only a few simulations are run, making it a good choice for surrogate modelling for building performance studies in general.

## References

[1] Vu K.K., D’Ambrosio C., Hamadi Y., Liberti L., Surrogate-based methods for black-box optimization, Int Trans Oper Res 24 (2017) 393–424. doi: 10.1111/itor.12292

[2] Westermann P., Evins R., Surrogate modelling for sustainable building design—A review, Energ Build 198 (2019) 170–186. doi: 10.1016/j.enbuild.2019.05.057

[3] Roman N.D., Bre F., Fachinotti V.D., Lamberts R., Application and characterization of metamodels based on artificial neural networks for building performance simulation: A systematic review, Energ Build 217 (2020) 109972. doi: 10.1016/j.enbuild.2020.109972

[4] Lu C.J., Li S.H., Lu Z.J., Building energy prediction using artificial neural networks: A literature survey, Energ Build 262 (2022) 111718. doi: 10.1016/j.enbuild.2021.111718

[5] Wang Z.Y., Srinivasan R.S., A review of artificial intelligence based building energy use prediction: Contrasting the capabilities of single and ensemble prediction models, Renew Sustain Energy Rev 75 (2017) 796–808. doi: 10.1016/j.rser.2016.10.079

[6] Friedman J.H., Greedy function approximation: a gradient boosting machine, Ann Stat 29 (2001), 1189–1232. doi: 10.1214/aos/1013203451

[7] Papadopoulos S., Azar E., Woon W.L., Kontokosta C.E., Evaluation of tree-based ensemble learning algorithms for building energy performance estimation, J Build Perform Simu 11 (2018) 322–332. doi: 10.1080/19401493.2017.1354919

[8] Tsanas A., Xifara A., Accurate quantitave estimation of energy performance of residential buildings using statistical machine learning tools, Energ Build 49 (2012) 560–567. doi: 10.1016/j.enbuild.2012.03.003

[9] Miller C., Arjunan P., Kathirgamanathan A., Fu C., Roth J., Park J.Y., et al, The ASHRAE Great Energy Predictor III competition: Overview and results, Sci Technol Built En 26 (2020) 1427–1447. doi: 10.1080/23744731.2020.1795514

[10] T.Q. Chen, C. Guestrin, XGBoost: A Scalable Tree Boosting System. In: Proc. 22nd ACM SIGKDD International Conference on Knowledge Discovery and Data Mining, San Francisco, USA, 2016, pp. 785–794.

[11] D. Nielsen, Tree boosting with XGBoost: Why does XGBoost wins “every” machine learning competition?, Master thesis, Norwegian University of Science and Technology, Trondheim, Norway, 2016.

[12] Fan C., Xiao F., Zhao Y., A short-term building cooling load prediction method using deep learning algorithms, Appl Energy 195 (2017) 222–233. doi: 10.1016/j.apenergy.2017.03.064

[13] Touzani S., Granderson J., Fernandes S., Gradient boosting machine for modeling the energy consumption of commercial buildings, Energ Build 158 (2018) 1533–1543. doi: 10.1016/j.enbuild.2017.11.039

[14] Wang R., Lu S.L., Li Q.P., Multi-criteria comprehensive study on predictive algorithm of hourly heating energy consumption for residential buildings, Sustain Cities Soc 49 (2019) 101623. doi: 10.1016/j.scs.2019.101623

[15] Song Z., Cao S.L., Yang H.X., Assessment of solar radiation resource and photovoltaic power potential across China based on optimized interpretable machine learning model and GIS-based approaches, Appl Energy 339 (2023) 121005. doi: 10.1016/j.apenergy.2023.121005

[16] Robinson C., Dilkina B., Hubbs J., Zhang W.W., Guhathakurta S., Brown M.A., et al, Machine learning approaches for estimating commercial building energy consumption, Appl Energy 208 (2017) 889–904. doi: 10.1016/j.apenergy.2017.09.060

[17] Crombecq K., Laermans E., Dhaene T., Efficient space-filling and non-collapsing sequential design strategies for simulation-based modeling, Eur J Oper Res 214 (2011), 683–696. doi: 10.1016/j.ejor.2011.05.032

[18] S. Surjanović, D. Bingham, Virtual library of simulation experiments: test functions and datasets, available at www.sfu.ca/~ssurjano, accessed Aug 14, 2024.

[19] Stevanović S., Stevanović D., Optimisation of curvilinear external shading of windows in cellular offices, PLoS ONE 13 (2018), e0203575. doi: 10.1371/journal.pone.0203575 30192845 PMC6128593

[20] Stevanović S., Stevanović D., Dehmer M., On optimal and near-optimal shapes of external shading of windows in apartment buildings, PLoS ONE 14 (2019), e0212710. doi: 10.1371/journal.pone.0212710 30817800 PMC6394916

[21] Office of Energy Efficiency & Renewable Energy, Prototype building models, available at energycodes.gov/prototype-building-models, accessed Dec 10, 2023.

[22] Santosh Philip, Welcome to eppy’s documentation, available at eppy.readthedocs.io/en/latest/, accessed Dec 5, 2023.

[23] scikit-optimize: Sequential model-based optimization in Python, available at scikit-optimize.github.io, accessed Dec 15, 2023.

[24] SUrrogate MOdeling Lab, SED Toolbox, available at sumo.intec.ugent.be/home/software/sed, accessed Dec 15, 2023.

[25] Fuhg J.H., Fau A., Nackenhorst U., State-of-the-Art and Comparative Review of Adaptive Sampling Methods for Kriging, Archives of Computational Methods in Engineering 28 (2021), 2689–2747. doi: 10.1007/s11831-020-09474-6

[26] J.H. Fuhg, Matlab Implementation Of State-Of-The-Art Adapative Techniques for Ordinary Kriging, available at github.com/FuhgJan/StateOfTheArtAdaptiveSampling, accessed Dec 15, 2023.

[27] S. Stevanović, H. Dashti, M. Milošević, S. Al-Yakoob, D. Stevanović, Python project for studying overhang surrogate models, available at github.com/dragance106/overhang-surrogates, accessed Jan 5, 2024.

[28] A. Paszke, S. Gross, F. Massa, A. Lerer, J. Bradbury, G. Chanan, et al, PyTorch: An Imperative Style, High-Performance Deep Learning Library, In: Proc NeurIPS 2019–Advances in Neural Information Processing Systems 32 (eds. H. Wallach, H. Larochelle, A. Beygelzimer, F. d’ Alché-Buc, E. Fox, R. Garnett), Vancouver, Canada, 2019, December 8–14, Curran Associates, Red Hook, NY, USA, 2019, pp. 8024–8035, available at papers.neurips.cc/paper/9015-pytorch-an-imperative-style-high-performance-deep-learning-library.pdf, accessed Jan 10, 2024.

[29] S. Stevanović, H. Dashti, M. Milošević, S. Al-Yakoob, D. Stevanović, Simulation data for the office cell building energy model with the attached overhang, available at zenodo.org/record/8169707, accessed Jan 5, 2024.

[30] R.H. Henninger, M.J. Witte, EnergyPlus Testing with ASHRAE 1052-RP Toolkit—Building Fabric Analytical Tests, Office of Energy Efficiency & Renewable Energy, 2015, available at energyplus.net/assets/nrel_custom/eplus_files/current_testing_reports/ASHRAE1052RP-8.3.0-b45b06b780.pdf, accessed Jan 23, 2024.

